# Clinical utility of lipoprotein(a) and the ApoB/ApoA-I ratio in the risk assessment of acute coronary syndrome: from single biomarkers to integrated risk stratification

**DOI:** 10.3389/fcvm.2026.1937328

**Published:** 2026-09-01

**Authors:** Zuqiang Lu, Pankui Li, Ruirui Li, Xiangqian Guo, Shuangshuang Li, Jing Wang

**Affiliations:** 1 The First School of Clinical Medicine, Ningxia Medical University, Yinchuan, China; 2 Department of Cardiology, Xi'an Ninth Hospital, Xi'an, China; 3 Department of Respiratory Medicine, Third Clinical Medical College of Ningxia Medical University, People's Hospital of Ningxia Hui Autonomous Region, Yinchuan, China

**Keywords:** acute coronary syndrome, apolipoprotein B/apolipoprotein A-I ratio, atherosclerosis, lipoprotein(a), risk stratification

## Abstract

Acute coronary syndrome (ACS) is a critical clinical emergency resulting from the rupture or erosion of coronary atherosclerotic plaques and subsequent thrombosis. Its pathogenesis is driven by a complex interplay of dysregulated lipid metabolism, inflammatory responses, thrombotic processes, and genetic susceptibility. Although traditional lipid parameters are widely utilized in ACS risk assessment, they remain limited in their ability to account for residual cardiovascular risk, inherited lipid abnormalities, and the overall atherosclerotic burden. Lipoprotein(a) [Lp(a)], an independent, genetically determined cardiovascular risk factor, exhibits distinctive pro-atherogenic, pro-inflammatory, and pro-thrombotic properties. In parallel, the apolipoprotein B/apolipoprotein A-I (ApoB/ApoA-I) ratio reflects the equilibrium between atherogenic lipoprotein particles and anti-atherogenic lipoprotein functions, providing a more comprehensive measure of lipid-related metabolic risk. Recent evidence has highlighted the value of both biomarkers in assessing coronary lesion severity, plaque instability, and prognosis in ACS. This review synthesizes current findings regarding the pathophysiological basis, clinical utility, and potential for combined assessment of Lp(a) and the ApoB/ApoA-I ratio, while critically discussing the limitations of existing research. Future investigations should shift from univariate biomarker analysis toward multi-marker integration, risk reclassification, genetic stratification, and validation of targeted interventions to advance precision risk assessment and individualized management of ACS.

## 1 Introduction

Acute coronary syndrome (ACS) represents a spectrum of clinical syndromes pathologically characterized by the rupture or erosion of coronary atherosclerotic plaques, resulting in complete or incomplete thrombotic occlusion. This syndrome encompasses unstable angina (UA), ST-segment elevation myocardial infarction (STEMI), and non-ST-segment elevation myocardial infarction (NSTEMI). As one of the most severe clinical manifestations of coronary atherosclerotic heart disease, ACS significantly compromises both patients' quality of life and survival outcomes (1). The high prevalence of ACS not only poses significant health risks to individuals but also exerts a substantial burden on public health systems. Consequently, early screening and effective management of cardiovascular risk factors in this population have become imperative. While low-density lipoprotein cholesterol (LDL-C) remains the cornerstone of cardiovascular risk assessment and lipid-lowering therapy, clinical practice reveals that a considerable proportion of patients still develop ACS or experience recurrent major adverse cardiovascular events (MACE) despite achieving guideline-recommended LDL-C targets (2). This observation underscores a critical limitation of traditional lipid parameters: they are insufficient to fully account for the residual cardiovascular risk in ACS. Consequently, there is an urgent need to incorporate novel biomarkers that can comprehensively reflect lipoprotein particle number, hereditary dyslipidemia, plaque instability, and prothrombotic propensity.

Lipoprotein(a) [Lp(a)] and the apolipoprotein B/apolipoprotein A-I (ApoB/ApoA-I) ratio represent two distinct yet complementary dimensions of cardiovascular risk. Lp(a) is a plasma lipoprotein structurally similar to low-density lipoprotein (LDL), but it is uniquely characterized by the surface-bound apolipoprotein(a) [Apo(a)] (3), which confers genetically determined pro-inflammatory and prothrombotic properties (4). In contrast, ApoB and ApoA-I serve as critical lipoprotein constituents: ApoB is the primary structural protein of all atherogenic particles, whereas ApoA-I is the major component of high-density lipoprotein (HDL) (5). The ApoB/ApoA-I ratio effectively captures the imbalance between atherogenic particle burden and HDL-mediated protection, thereby serving as a robust marker for assessing atherosclerotic risk (6). Consequently, these two biomarkers may complement traditional lipid parameters by providing novel insights into different pathophysiological pathways, offering new perspectives for risk stratification, prognostic evaluation, and individualized management in patients with acute coronary syndrome (ACS). Nevertheless, existing literature has predominantly focused on the association of individual biomarkers with clinical outcomes, and there remains insufficient evidence to determine whether their combined application truly enhances predictive performance.

## 2 Literature search and review methodology

This article is a structured narrative review aimed at synthesizing and critically evaluating the literature regarding the clinical utility of Lp(a) and the ApoB/ApoA-I ratio in the context of ACS. We conducted a comprehensive literature search across PubMed, Web of Science, Embase, the China National Knowledge Infrastructure (CNKI), and the Wanfang Database, covering records from the inception of each database through July 14, 2026. Search terms included: “lipoprotein(a),” “Lp(a),” “ApoB/ApoA-I ratio,” “apolipoprotein B,” “apolipoprotein A-I,” “acute coronary syndrome,” “coronary artery disease,” “atherosclerosis,” and “major adverse cardiovascular events.”

**Figure 1:**
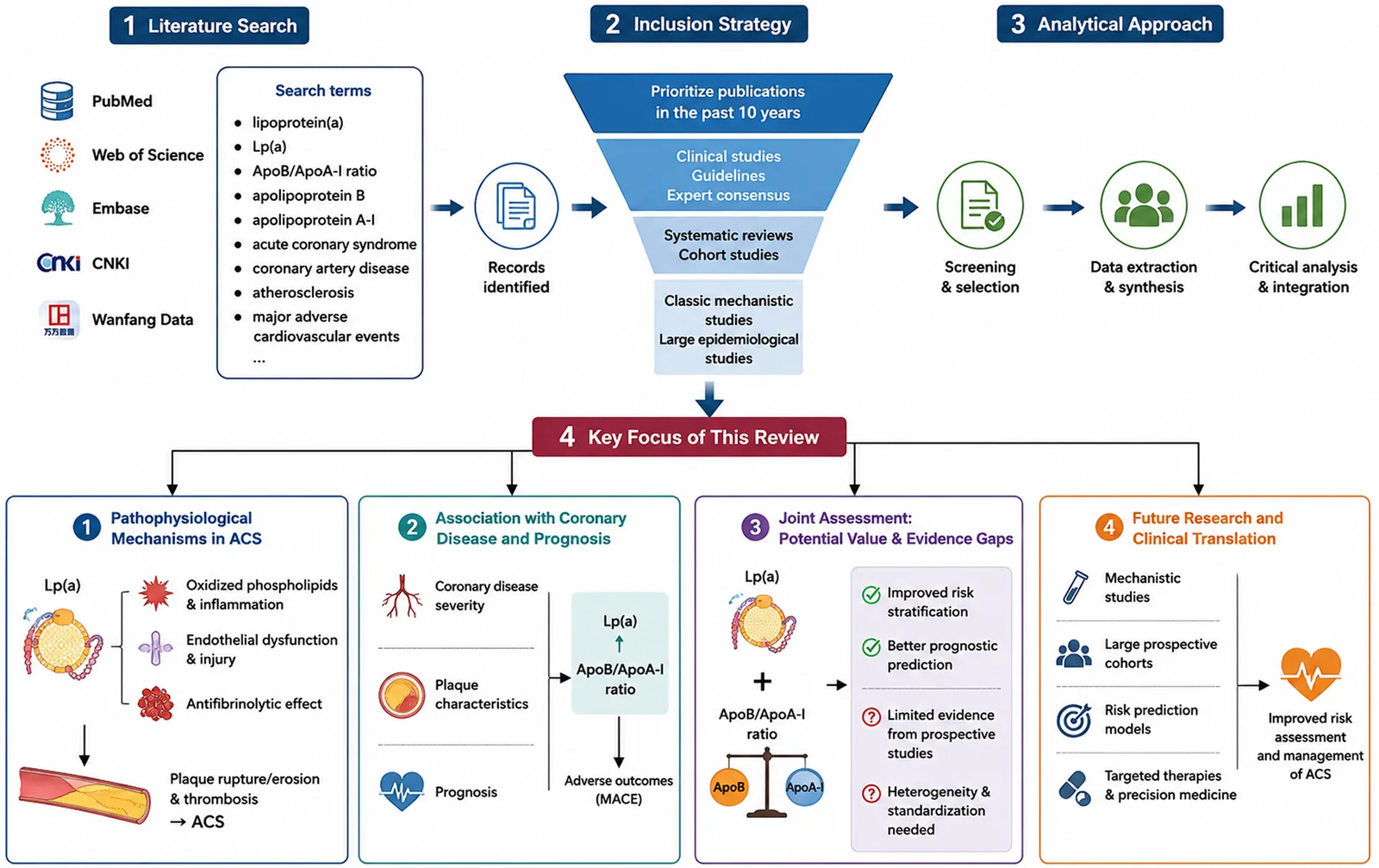
Literature-search and evidence-selection framework for this narrative review.

The study selection process was conducted in two sequential stages. First, titles and abstracts were screened to identify potentially eligible records. Subsequently, full-text evaluations were performed to finalize inclusion based on predefined criteria. Priority was given to high-level evidence relevant to the scope of this review, including: (1) clinical trials and prospective cohort studies; (2) meta-analyses and systematic reviews; (3) seminal mechanistic and epidemiological studies; and (4) clinical practice guidelines and expert consensus statements. Conversely, single-case reports, conference abstracts lacking full-text data, and studies with significant methodological limitations were excluded. Any uncertainties regarding eligibility were resolved through discussion and consensus among the author team. The literature search and evidence selection framework is illustrated in Figure 1.

To ensure rigorous evidence synthesis, we categorized the included studies based on their populations. Evidence derived from cohorts explicitly enrolling ACS patients (e.g., STEMI, NSTE-ACS) is classified as ‘direct ACS evidence.’ Evidence from stable coronary artery disease (CAD), broader atherosclerotic cardiovascular disease (ASCVD), or general community cohorts is presented as ‘indirect or supportive evidence,’ providing necessary biological and prognostic context without being interpreted as definitive evidence for ACS management.

## 3 Pathophysiological basis and clinical significance of lipoprotein(a) in acute coronary syndrome

Lp(a) is a distinct and genetically determined plasma lipoprotein first identified in 1963 by the Norwegian geneticist Arne Berg during investigations into the genetic heterogeneity of LDL (7). Structurally, Lp(a) consists of two primary components: ApoB and apolipoprotein(a) [Apo(a)] (8). The seminal discovery by McLean et al. revealed that Lp(a) shares significant structural homology with plasminogen (9), which confers unique biological functions to Lp(a). Beyond its role in lipid deposition, Lp(a) enhances thrombotic propensity by interfering with fibrinolytic processes. Furthermore, the structural diversity and genetic polymorphisms of Apo(a) significantly modulate circulating Lp(a) concentrations (10), and these variations are linked to the risk of cardiovascular disease (11). Unlike LDL-C, Lp(a) levels are predominantly determined by genetic factors and remain largely refractory to lifestyle modifications and conventional lipid-lowering therapies (12, 13). These distinctive characteristics establish Lp(a) as a critical biomarker for the assessment of residual cardiovascular risk.

Mechanistically, Lp(a) drives the pathogenesis of ACS through multiple interconnected pathways:

(1) Antifibrinolytic and Prothrombotic Effects: The structural mimicry between Apo(a) and plasminogen enables Lp(a) to competitively inhibit fibrinolysis, thereby promoting thrombus formation (13, 14). Elevated Lp(a) levels are further associated with increased thrombus stability, which exacerbates the risk of acute cardiovascular events (15).
(2) Arterial Lipid Deposition and Atherosclerosis: Lp(a) particles preferentially accumulate within the arterial wall, facilitating lipid-rich deposition that initiates and accelerates the progression of atherosclerosis (16).
(3) Inflammatory Cascade and Endothelial Dysfunction: Intra-arterial Lp(a) deposition triggers localized inflammatory responses (17). Additionally, Lp(a) may augment the levels of oxidized LDL (ox-LDL), promoting endothelial dysfunction and inflammatory signaling, which collectively facilitate atherosclerotic plaque formation and destabilization (18).

**Figure 2:**
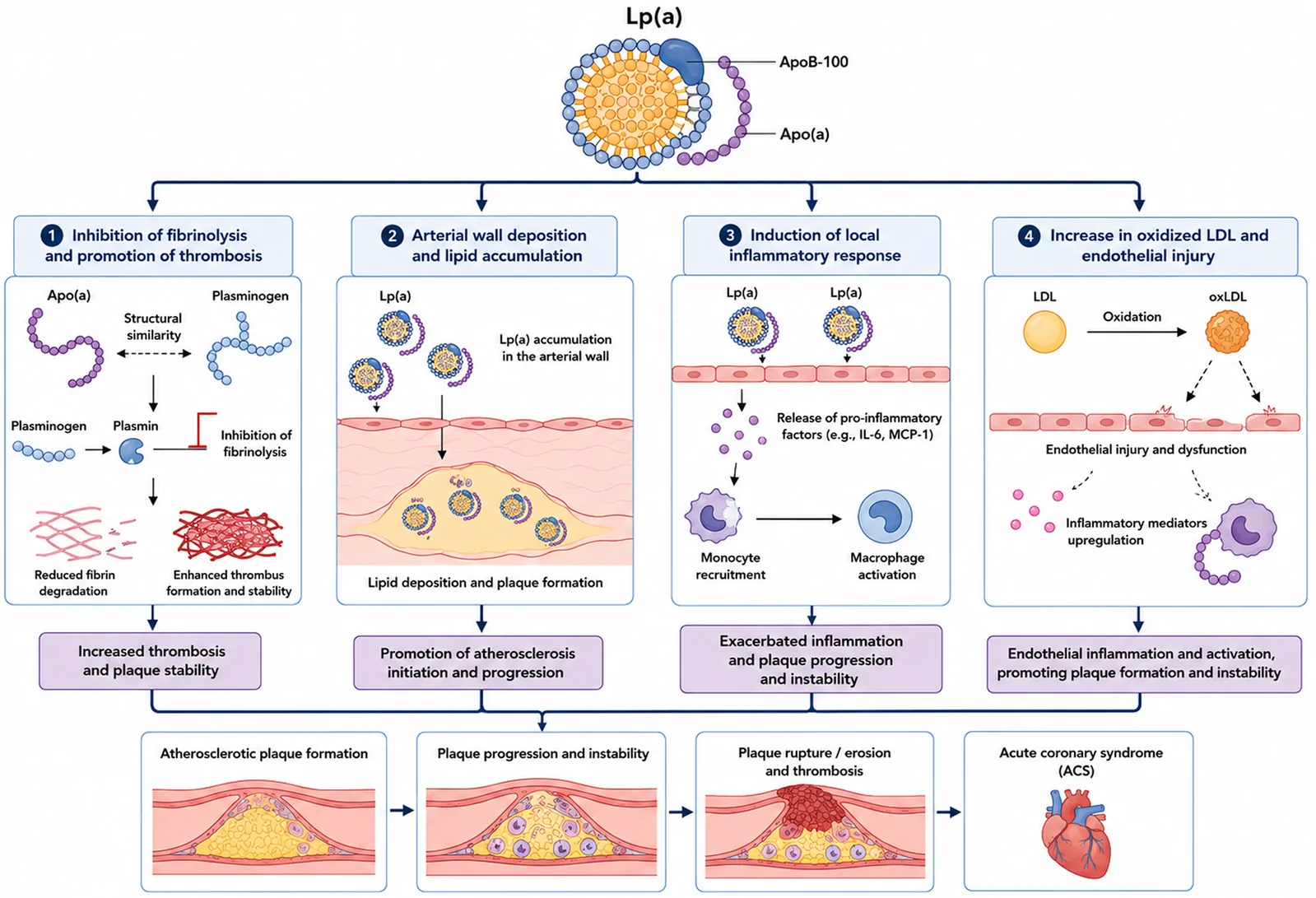
Schematic diagram of molecular mechanisms mediated by Lp**(a)** in the development of acute coronary syndrome.

These multiple pathophysiological pathways constitute a comprehensive mechanistic network driving ACS, the complex interdependencies of which are illustrated in Figure 2.

A substantial body of epidemiological evidence has established that elevated Lp(a) levels are closely associated with an increased risk of various atherosclerotic cardiovascular diseases, including coronary artery disease, peripheral artery disease, and aortic valve stenosis (19). However, when evaluating its clinical significance, it is essential to clearly distinguish evidence derived directly from ACS cohorts from that obtained in broader cardiovascular populations. Direct evidence from ACS-specific cohorts provides insights into the clinical relevance of Lp(a) in the acute setting. For example, at the 2023 European Society of Cardiology (ESC) Congress, a study from Poland investigated the relationship between elevated Lp(a) and the severity of coronary atherosclerosis in patients with ACS (20). This study enrolled 74 hospitalized patients, who were stratified according to their Lp(a) levels [Lp(a) < 75 nmol/L (*n* = 53) vs. Lp(a) ≥ 75 nmol/L (*n* = 21)]. Comparative analysis revealed a higher prevalence of hypertension and hyperlipidemia among patients with elevated Lp(a) levels. Both univariable and multivariable regression analyses demonstrated that elevated Lp(a) was independently associated with an increased risk of triple-vessel coronary artery disease and multivessel involvement. Furthermore, the study indicated that Lp(a) ≥ 75 nmol/L was closely correlated with the extent and severity of coronary artery lesions.

However, current evidence regarding the prognostic value of Lp(a) following ACS remains inconsistent. While some studies have shown that elevated Lp(a) correlates with a greater atherosclerotic burden and a higher risk of cardiovascular events (21), Roth et al. did not find a significant association between Lp(a) and post-ACS survival (22), suggesting that its prognostic value may be influenced by specific ACS phenotypes and other clinical variables. The inconsistencies in previous findings may stem from heterogeneity across studies in population characteristics, sample sizes, definitions of clinical endpoints, follow-up durations, Lp(a) threshold settings, assay methods, and baseline treatment regimens (e.g., secondary prevention strategies). Therefore, although Lp(a) may provide information on residual cardiovascular risk, its independent and incremental prognostic value in the ACS population requires further validation through adequately powered prospective studies.

Evidence from broader cardiovascular populations offers complementary, indirect support for the role of Lp(a). Data from the UK Biobank have shown that in patients with incident ASCVD, Lp(a) ≥ 150 nmol/L is associated with a higher risk of MACE, irrespective of LDL-C levels (23). Similarly, a large cohort study by Berman et al., involving individuals with and without baseline ASCVD, confirmed that elevated Lp(a) is associated with an increased risk of MACE (24). In both ACS-specific and broader cardiovascular populations, this association often persists among patients with lower LDL-C levels or those receiving statin therapy, indicating that Lp(a) captures a component of residual cardiovascular risk that is not fully addressed by traditional lipid parameters (25–27). In summary, although elevated Lp(a) levels are biologically linked to atherosclerotic disease, their clinical value in the context of ACS should be comprehensively interpreted by integrating ACS-specific findings with broader epidemiological evidence.

However, several limitations in the current evidence base should be acknowledged. First, Lp(a) thresholds vary substantially across studies, and the lack of a standardized conversion between mg/dL and nmol/L limits the comparability of findings. Second, Lp(a) levels exhibit significant ethnic heterogeneity, and the applicability of a universal cutoff remains a subject of scientific debate. Third, the majority of existing evidence is derived from observational studies; while genetic data support a causal link between Lp(a) and atherosclerotic cardiovascular disease, the predictive utility of Lp(a) specifically in ACS cohorts requires further prospective validation. Fourth, evidence remains insufficient to confirm that Lp(a)-lowering therapy alone definitively reduces recurrent cardiovascular events in ACS patients, and the clinical benefits of targeted interventions await results from large-scale outcome trials.

## 4 Clinical value of the ApoB/ApoA-I ratio in ACS

Apolipoproteins (Apos) are the primary structural proteins of lipoprotein particles. They guide lipid transport and interact with specific receptors to facilitate lipid uptake and deposition into tissues, thereby playing a central role in cholesterol metabolism (28). ApoB is the principal protein involved in cholesterol transport and is present in all atherogenic lipoprotein particles, including LDL and very-low-density lipoprotein (VLDL) (29). Since each of these particles typically contains a single ApoB molecule, ApoB concentration provides a more direct reflection of the total number of atherogenic particles. ApoB plays a pivotal role in lipid transport by binding to cholesterol receptors on cell membranes, thereby promoting cholesterol uptake (30). Consequently, ApoB concentration is directly proportional to the total number of circulating cholesterol carriers, serving as a direct indicator for cardiovascular risk assessment. In contrast, ApoA-I is the major protein constituent of HDL (31). It plays a crucial role in reverse cholesterol transport (RCT), facilitating the efflux of cholesterol from peripheral tissues back to the liver, thereby reducing arterial cholesterol accumulation and exerting a cardioprotective effect (32). An elevated ApoB/ApoA-I ratio typically signifies a higher burden of atherogenic risk factors, reflecting a relative predominance of pro-atherogenic LDL particles coupled with diminished HDL-mediated protective capacity (33). Therefore, the ApoB/ApoA-I ratio provides a comprehensive assessment of the balance between cholesterol transport and metabolic homeostasis (34), establishing itself as a critical metric for cardiovascular disease risk evaluation (35).

Compared with traditional cholesterol parameters, the ApoB/ApoA-I ratio offers distinct advantages. While LDL-C measures the mass of cholesterol carried within particles, ApoB more accurately approximates the actual number of lipoprotein particles (36). In clinical scenarios involving diabetes, metabolic syndrome, hypertriglyceridemia, or an abundance of small, dense LDL (sdLDL) particles, LDL-C may underestimate the true atherosclerotic risk (37). In such instances, the ApoB/ApoA-I ratio provides incremental diagnostic value (38). In ACS patients, a higher ApoB/ApoA-I ratio is often indicative of a greater atherosclerotic burden, more complex coronary lesions, and a poorer prognosis.

Evidence regarding the ApoB/ApoA-I ratio should be interpreted according to the source population. In the ACS-specific setting, Wang et al. reported that an elevated ApoB/ApoA-I ratio is strongly associated with adverse outcomes, with higher ratios predicting worse clinical prognoses (39). In contrast, evidence derived from broader cardiovascular populations highlights the general utility of this ratio. Jiang et al. demonstrated superior discriminatory performance for coronary artery disease and clinical phenotypes requiring revascularization in a broad cohort of suspected coronary disease patients (40). Similarly, a multi-ethnic cohort study from the United States revealed its predictive value for coronary heart disease mortality (41), and it serves as a sensitive marker for risk identification in individuals with normal cholesterol levels but high overall cardiovascular risk (42). While these findings from broader populations provide robust support for the ApoB/ApoA-I ratio as a cardiovascular marker, they should be considered indirect evidence when extrapolated to ACS risk stratification. Overall, the ApoB/ApoA-I ratio may serve as a complementary marker in ACS management, although its incremental prognostic value requires further validation in dedicated, large-scale ACS cohorts.

However, the clinical application of the ApoB/ApoA-I ratio remains limited. First, there is a lack of a universally accepted standard, with significant variability in high-risk cut-off points across studies. Second, ApoA-I reflects not only HDL quantity but also its functional capacity (43); current routine assays often fail to adequately evaluate the anti-inflammatory, antioxidant, and cholesterol efflux capacities of HDL. Third, the ApoB/ApoA-I ratio is highly correlated with LDL-C, non-HDL-C, triglycerides, and other metabolic markers (44), and its independent predictive value in multivariate models requires further validation. Therefore, this ratio is best utilized as a complementary adjunct to, rather than a total replacement for, traditional lipid assessment.

## 5 Combined assessment of Lp(a) and ApoB/ApoA-I ratio: theoretical framework

The concurrent elevation of both Lp(a) and ApoB/ApoA-I ratio typically signifies substantially heightened cardiovascular event risk in individual patients (45). Whereas patients with elevated Lp(a) alone face augmented atherosclerotic complications, those with elevated ApoB/ApoA-I ratio may harbor underlying cholesterol metabolic dysregulation. The synergistic application of both indices derives inherent validity from their capacity to capture distinct mechanistic pathways underlying ACS pathogenesis. Specifically, the ApoB/ApoA-I ratio primarily reflects the cumulative burden of atherogenic lipoprotein particles and systemic lipid metabolic imbalance, thereby indicating enhanced risk for atherosclerotic plaque initiation and progression. In contrast, Lp(a) further embodies hereditary dyslipidemia, oxidized phospholipid accumulation, systemic inflammatory response, and prothrombotic predisposition. Conceptually, the ApoB/ApoA-I ratio may be viewed as capturing “atherosclerotic plaque formation burden" (43), whereas Lp(a) more specifically reflects “plaque instability and thrombotic event propensity" (46, 47). When applied in combination, these complementary biomarkers more comprehensively characterize the complete spectrum of ACS risk than either parameter alone.

**Figure 3:**
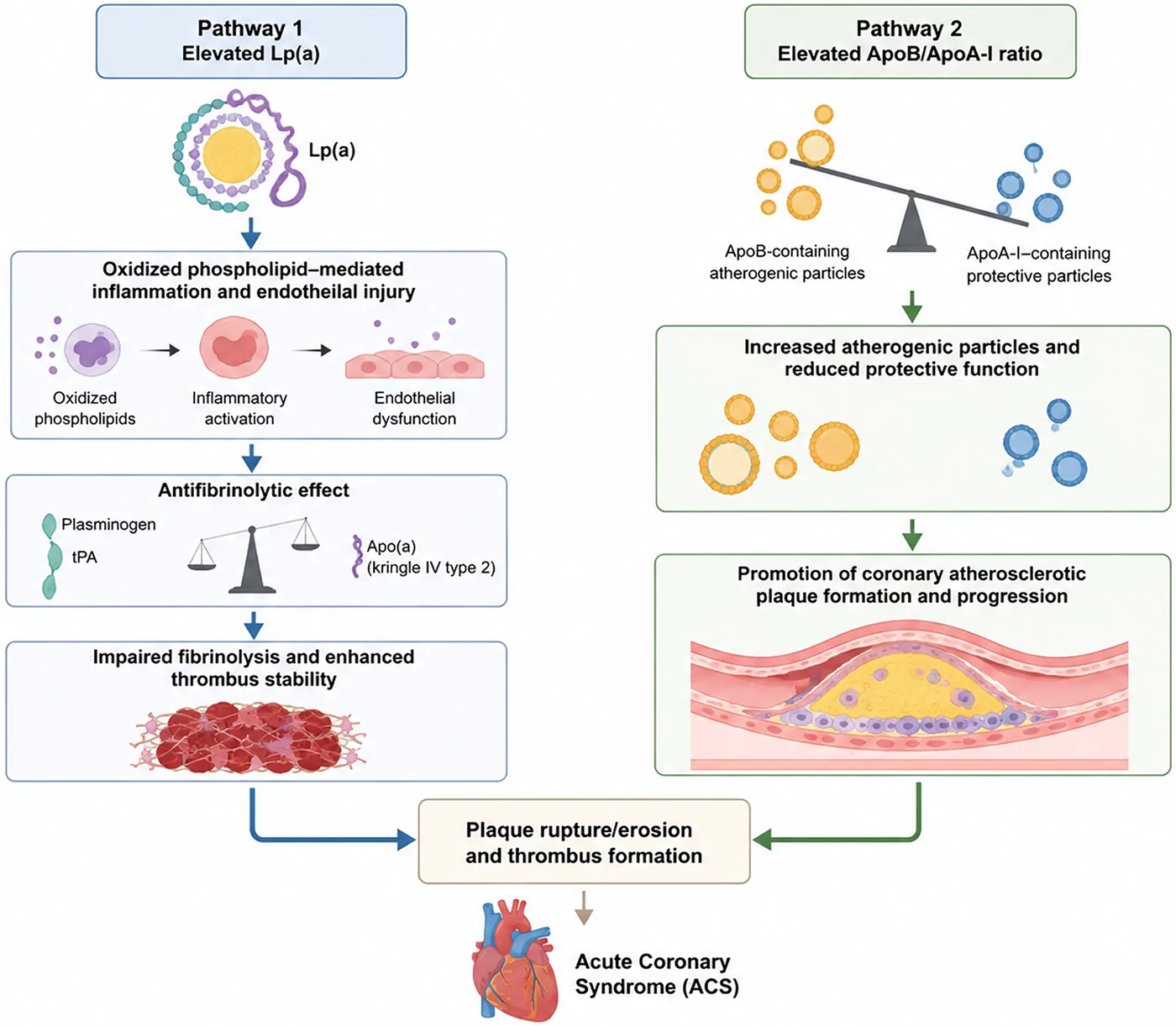
Dual-pathway mechanistic model of ACS pathogenesis mediated by the combined effects of Lp**(a)** and ApoB/ApoA-I ratio.

This conceptual framework can be understood as a dual-pathway mechanistic model. Pathway 1 (Plaque Formation Pathway): An elevated ApoB/ApoA-I ratio reflects increased accumulation of atherogenic lipoprotein particles and insufficient cardioprotective lipoprotein function, thereby promoting coronary atherosclerotic plaque formation and progression (43, 48). This pathway represents the “atherosclerotic plaque burden” dimension of cardiovascular risk. Pathway 2 (Plaque Destabilization and Thrombosis Pathway): Elevated Lp(a) exerts pathogenic effects primarily through oxidized phospholipid-mediated inflammation, endothelial injury, and antifibrinolytic mechanisms, rendering pre-existing atherosclerotic plaques more susceptible to rupture, superficial erosion, and subsequent thrombotic occlusion (49, 50). This pathway embodies the “plaque instability and thrombotic predisposition” dimension of ACS risk. Synergistic Risk Integration: When both biomarkers are simultaneously elevated, patients theoretically harbor concurrent high atherosclerotic plaque burden and pronounced prothrombotic tendency, thereby constituting an exceptionally high-risk phenotype (51). The complementary pathophysiological mechanisms of these two biomarkers work synergistically to compound overall ACS risk. Detailed mechanistic pathways are illustrated in Figure 3.

However, the current evidence base for the combined assessment of these two biomarkers remains insufficient. Most existing literature discusses the relationship of either Lp(a) or the ApoB/ApoA-I ratio with ACS independently; studies genuinely integrating both biomarkers into a unified predictive model and rigorously evaluating their incremental predictive value remain scarce. Future investigations should move beyond merely reporting correlations or odds ratios; instead, they should employ rigorous statistical comparisons of combined models versus traditional models, specifically evaluating changes in C-statistic, Net Reclassification Improvement (NRI), Integrated Discrimination Improvement (IDI), and calibration. Furthermore, it is essential to explore whether this combined approach provides additional clinical value beyond established risk stratification tools, such as the GRACE score, SYNTAX score, or conventional lipid-based models. Such evidence would facilitate more accurate identification of high-risk individuals, enable better assessment of ACS severity and prognosis, and ultimately provide clinicians with individualized risk assessment and treatment strategies tailored to patient phenotype. Concurrently, in the context of pharmacological interventions and lifestyle modifications, the dual assessment of these markers may enhance the clinician's understanding of a patient's cardiovascular status, provide a robust evidentiary basis for clinical decision-making, and improve the efficacy of early detection and intervention, thereby optimizing outcomes in ACS patients.

## 6 Major evidence gaps compared with existing literature

**Figure 4:**
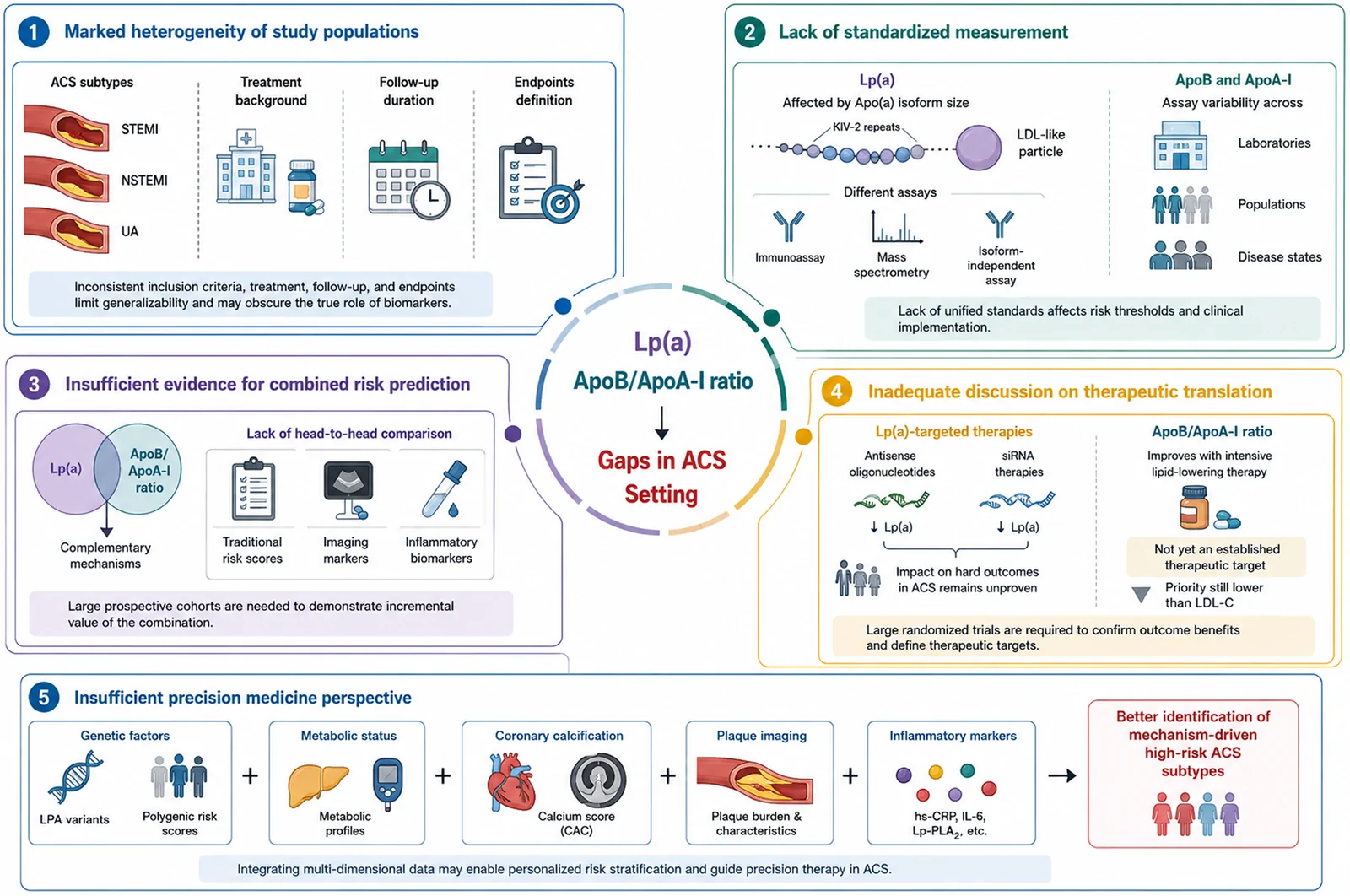
Conceptual framework of current research limitations in the combined assessment of Lp**(a)** and the ApoB/ApoA-I ratio in ACS.

Extant literature has adequately substantiated that Lp(a) and the ApoB/ApoA-I ratio are independently associated with the risk of atherosclerotic cardiovascular disease; however, significant evidence gaps persist specifically in the context of ACS. The following five major limitations merit consideration:

1. Study Population Heterogeneity: Marked heterogeneity characterizes study populations across existing investigations. The inclusion of diverse ACS phenotypes (STEMI, NSTEMI, and UA), therapeutic contexts, and endpoint definitions restricts the generalizability of conclusions. Failure to perform stratified analyses risks masking the true prognostic impact of these biomarkers.
2. Lack of Standardized Detection Methods: Consensus regarding standardized detection methodology remains incomplete. Lp(a) measurement is substantially confounded by apo(a) isoform size polymorphisms, leading to potential discrepancies between assay platforms. Furthermore, the lack of harmonized reference ranges for ApoB and ApoA-I across diverse clinical settings hinders the establishment of universal risk thresholds (52).
3. Insufficient Evidence for Combined Risk Prediction: While the theoretical complementarity of these markers is compelling, large-scale prospective cohort studies demonstrating that their combined application significantly improves ACS risk prediction are lacking. Notably, direct head-to-head comparisons with established risk stratification instruments and multimodal imaging markers are currently absent.
4. Inadequate Translation to Clinical Therapeutics: Emerging Lp(a)-targeted therapies (e.g., ASOs, siRNA) show substantial potential (53), yet their ability to reduce hard clinical endpoints in ACS patients requires validation through large-scale cardiovascular outcome trials. Similarly, the status of the ApoB/ApoA-I ratio as an independent therapeutic target remains secondary to LDL-C.
5. Insufficient Integration of Precision Medicine Principles: Lp(a) levels are predominantly genetically determined, whereas the ApoB/ApoA-I ratio reflects metabolic state. Future investigations incorporating *LPA* gene variants, polygenic risk scores, and plaque imaging characteristics may enable more accurate identification of high-risk ACS phenotypes driven by distinct underlying mechanisms. A comprehensive summary of these limitations is presented in Figure 4.

## 7 Clinical implications

In recent years, research on Lp(a) has advanced significantly, with mounting evidence elucidating how genetic variants influence its plasma levels and its role in cardiovascular disease (CVD). It should be noted that the evidence discussed in this section is derived primarily from broader CVD populations and early-phase clinical trials, rather than from ACS-specific cohorts. A recent study by Kamstrup et al. firmly established Lp(a) as an independent, genetically determined risk factor for a spectrum of cardiovascular diseases, including coronary artery disease, peripheral artery disease, ischemic stroke, and calcific aortic valve stenosis (54). Through extensive observational studies and genetic analyses, these findings have revealed a robust association between Lp(a) levels and cardiovascular risk. Notably, the distribution of Lp(a) varies substantially across different ethnic and geographic populations, underscoring the importance of incorporating Lp(a) assessment into routine clinical testing. Furthermore, novel therapeutic strategies targeting Lp(a) are currently under development (55). An early-phase clinical trial by Nissen et al.—not specific to ACS—demonstrated that the novel therapeutic agent lepodisiran achieved sustained reductions in serum Lp(a) concentrations among 48 participants with elevated Lp(a) levels (56). Although this evidence is derived mainly from broader populations, it provides important indirect support for risk management in patients with ACS.

Meanwhile, a review by Marcovina et al. highlighted that the ApoB/ApoA-I ratio is closely associated with atherosclerosis and may serve as a predictor of cardiovascular events (57). Yuan et al. further reported that, among patients with coronary artery disease undergoing percutaneous coronary intervention (PCI)—a cohort not restricted to ACS patients—the ApoB/ApoA-I ratio outperformed individual lipid parameters and the non-high-density lipoprotein cholesterol/high-density lipoprotein cholesterol (non-HDL-C/HDL-C) ratio in predicting total coronary occlusion (58). Furthermore, the AMORIS study evaluated the clinical utility of this ratio in cerebrovascular disease by following 98,722 men and 76,831 women for a mean of 10.3 years. The results showed that, compared with conventional lipid ratios [such as total cholesterol/HDL-C (TC/HDL-C) and HDL-C/LDL-C], the ApoB/ApoA-I ratio demonstrated greater predictive capacity for cerebrovascular disease and exhibited a linear association with stroke risk. Accordingly, the study identified the ApoB/ApoA-I ratio as a core indicator of cholesterol transport and cardiovascular risk, characterizing it as a highly specific marker of ischemic events (59). Collectively, these findings strongly support the value of this ratio in reflecting the overall atherosclerotic burden; however, caution is warranted when extrapolating these findings to patients with ACS, as its clinical significance as an ACS-specific prognostic marker requires validation in future studies.

In clinical practice, Lp(a) levels have emerged as a critical metric for cardiovascular risk assessment. While not yet a component of routine screening, Lp(a) testing significantly facilitates risk evaluation in high-risk patients or individuals with familial hypercholesterolemia (60, 61). Some medical institutions have already integrated Lp(a) into high-risk assessment protocols to guide the formulation of personalized treatment plans (15). Similarly, the ApoB/ApoA-I ratio is increasingly used to complement conventional cholesterol panels, providing a more holistic view of cardiovascular risk. Reflecting this expanding evidence base, clinical guidelines have been updated; for instance, the European Society of Cardiology (ESC) and European Atherosclerosis Society (EAS) guidelines recommend measuring ApoB as a surrogate marker for LDL particle number in specific scenarios. These guidelines emphasize the potential value of the ApoB/ApoA-I ratio in risk assessment, particularly when traditional lipid metrics are insufficient to capture the full risk profile (62).

For patients with ACS, the combined detection of these two markers should be viewed as a valuable supplement to traditional risk assessment, rather than a fully mature, standalone clinical decision-making tool. A more appropriate clinical positioning would be as an auxiliary indicator for refined risk stratification, integrated alongside conventional lipid profiles, cardiac biomarkers, inflammatory markers, coronary imaging, and clinical scores. For patients with concurrent elevations in both Lp(a) and the ApoB/ApoA-I ratio, clinicians should consider more intensive secondary prevention strategies, including more stringent LDL-C control, optimized lifestyle modifications, management of metabolic abnormalities, increased follow-up frequency, and consideration of future eligibility for Lp(a)-targeted therapies (63, 64).

## 8 Future research directions

Future investigations should prioritize advancement along the following strategic axes. First, large-scale, multicenter, prospective cohort studies are warranted to establish the independent predictive value of Lp(a) and the ApoB/ApoA-I ratio across distinct ACS phenotypes. Second, harmonization of measurement methodologies and reporting units is essential; in particular, Lp(a) assessment should employ standardized assays that are independent of Apo(a) isoform size, and risk-stratifying cutoff values applicable to diverse populations must be systematically defined. Third, comprehensive multivariable risk prediction models incorporating Lp(a), the ApoB/ApoA-I ratio, hs-CRP, coronary imaging parameters, and established clinical risk scores should be developed and validated. The clinical utility and incremental gain of these models should be rigorously evaluated using Net Reclassification Improvement (NRI), Integrated Discrimination Improvement (IDI), and decision curve analysis (DCA). Fourth, by leveraging genetic and precision medicine approaches, research should explore the complex interactions between *LPA* gene variants, ApoB-related metabolic phenotypes, and ACS risk. Fifth, as Lp(a)-targeted therapeutics enter clinical practice, priority should be given to clinical endpoint studies to determine whether pharmacological reduction of Lp(a) levels genuinely translates into reduced ACS recurrence and MACE.

## 9 Conclusion

**Figure 5:**
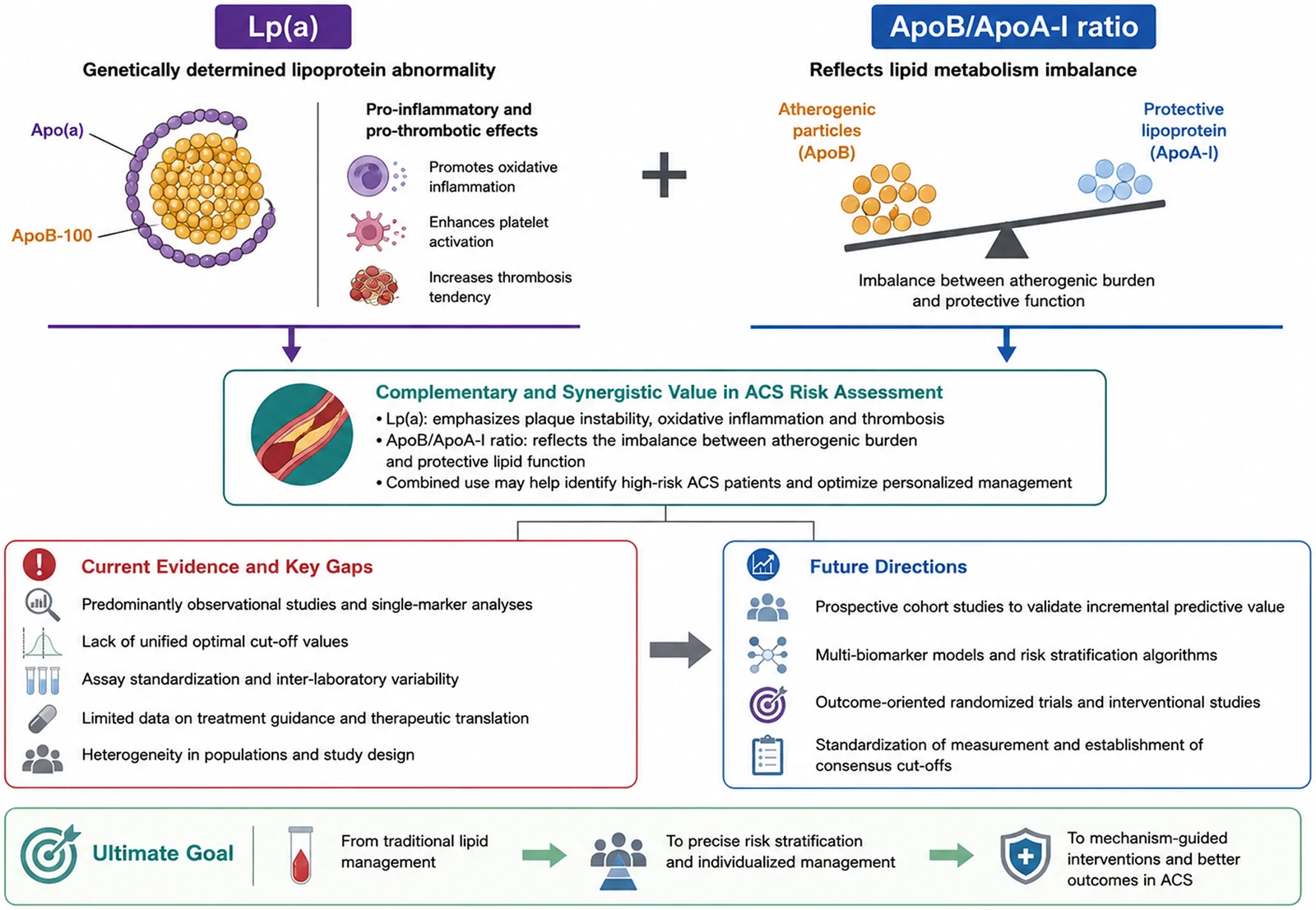
Comprehensive framework of Lp**(a)** and the ApoB/ApoA-I ratio for precision risk assessment in ACS.

The Lp(a) and ApoB/ApoA-I ratio provide a critical complement to traditional lipid parameters in ACS risk assessment by addressing hereditary lipoprotein abnormalities, pro-inflammatory and prothrombotic effects, and imbalances in lipid metabolism. Specifically, Lp(a) underscores plaque instability, oxidative inflammation, and thrombogenic propensity, while the ApoB/ApoA-I ratio reflects the disequilibrium between atherogenic particle burden and the functional capacity of protective lipoproteins. Their integrated application is theoretically robust and may facilitate the identification of high-risk ACS patients, thereby optimizing personalized management strategies. Nevertheless, existing evidence remains predominantly based on observational studies and single-marker analyses, with significant gaps persisting regarding their combined predictive value, optimal clinical cut-offs, standardized measurement protocols, and therapeutic translation. A comprehensive logical framework—encompassing the mechanistic basis, current research limitations, and future investigative priorities for these two biomarkers—is presented in Figure 5. Future efforts should leverage prospective research, multivariable marker models, and outcome-oriented trials to further validate their clinical utility, ultimately driving a paradigm shift in ACS risk assessment from conventional lipid management toward precision-based risk stratification and mechanism-driven interventions.
